# On the Mode in Which Coichicum Operates in the Cure of Acute Rheumatism

**Published:** 1835

**Authors:** 


III. *On the mode in which Colchicum operates in the cure of Acute Rheumatism.*—We are indebted to our persevering hebdominal brother, the Boston Medical and Surgical Journal, for a short paper from the Lancet, by Dr. A. T. Thompson, whom we recollect as an editor of the London Pharmacopœia, and who is a man of sound sense. The views presented by Dr. T. are so exactly in accordance with our own, that we are induced to present the article entire. It is as follows:

"The number of cases of acute rheumatism which has been admitted within the last twelve days, has again turned our attention to this disease: but I shall now direct your notice merely to one of these cases, not because the symptoms have displayed any peculiarity, but because the rapidity of the relief which followed the operation of the colchicum upon the alimentary canal; affords me an opportunity of explaining my opinions of the manner in which this valuable remedy usually produces beneficial effects in rheumatic inflammation. It has been customary to refer these effects of colchicum to its sedative or narcotic powers; an opinion which my experience prevents me from adopting; but in dissenting from this view of the influence of colchicum, I am anxious that I shall not be misunderstood as attempting to deny that it possesses sedative powers; although I have never seen these adequate to the production of permanent relief from pain in acute rheumatism. If a full dose of the wine of the seeds of the colchicum be administered after a moderate bleeding, it rarely fails to purge, and to maintain the reduction of the pulse caused by the abstraction of the blood. Under such circumstances it would be remarkable if there was no abatement of pain, if the system be brought under the influence of even a very moderate sedative. The purgative influence of the colchicum carries its sedative power, although weak, thus far, whilst it precludes the further employment of the lancet; and thus it seems to combine the advantages of purging, and the administration of narcotics. Now in taking this view of the influence of colchicum, I am convinced that it is never productive of much benefit in acute rheumatism, unless it purge freely. The evacuations are generally copious, liquid, and highly bilious, owing to the remedy, as a topical stimulant on the duodenum, exciting powerfully the orifices of the biliary duct, and causing a large flow of bile to the intestine, in the same manner as exciting the orifices of the salivary ducts in the mouth not only empties those glands, but augments their secreting function. The purgative effects of colchicum are sometimes excessive, and the consequent debility is great; but I have seen twelve or fourteen copious watery stools produced by a full dose of the drug, without any obvious debility resulting. If the lancet be not previously employed, the excitement present in the habit seems to resist both the purgative and sedative powers of colchicum; which, apparently, like those of some other remedies, require the habit to be brought into a certain condition, before they can operate in a salutary manner. It is on this account that I almost invariably, as you must have observed, order my patients with acute rheumatism to be bled, and the bleeding to be followed by a dose of calomel, tartar emetic, and opium, before prescribing colchicum. If the patient be of a plethoric habit, f. ʒj· or f. ʒiss. of the wine of the seeds may be given for a dose, six or eight hours after the administration of the pill; and this dose is to be repeated once in six hours until it begin to purge, when either the repetition should be deferred for twelve hours, or, if the effect be powerful, the medicine should be altogether discontinued. If febrile spmptoms recur, accompanied by pain, I have generally found the tartar emetic preferable to a return to full doses of the colchicum; but the influence of the antimonial is aided by small and frequently repeated doses of the colchicum. On the contrary, if the pulse remains soft, regular, and moderate in frequency; and the pains do not return, except perhaps in a slight degree in the evening; whilst the skin, also, remains cool, I have found that the decoction of yellow bark, acidulated with sulphuric acid, or the solution of the sulphate of quinia, acidulated in the same manner, tends more than any other means to secure the patient from a relapse, and to confirm the cure.

In making these remarks, it would be uncandid were I not to mention that most distressing effects have ocasionally followed the employment of large doses of colchicum; but these have either depended on the improper administration of the medicine, or on idiosyncracy. As a rule to guide you against the first error, you must recollect that colchicum ought never to be prescribed when the red or glazed state of the tongue indicates much irritation or a sub-acute inflammatory condition of the mucous membrane of the alimentary canal. In this case, moderate doses of the hydrocyanic acid and liquor potassæ with Dover's powder, aided by counter-irritants, may be employed to prepare the habit for the use of the colchicum; and even after the irritable state of the alimentary canal has been sufficiently subdued to admit of its use, the remedy ought to be prescribed in small doses, frequently repeated, instead of the full doses that may be administered under other circumstances. The deleterious influence of idiosyncracy can only be avoided by inquiring into the effects of the remedy when it has been taken at any prior time."

We have lately treated two cases of sub-acute rheumatism with the vinum colchici and laudanum with decided success. One of them, only, was under our immediate observation. Originally attended with intense inflammation, travelling from joint to joint, and resisting most copious depletion by the lancet, tartar-emetic, calomel, etc. we still found it, after the lapse of many weeks, an obstinate and painful affection. Under these circumstances, we gave the wine of colchicum in drachm and two drachm doses, in the evening with laudanum. It invariably purged the patient, and, both before and after that effect, afforded him signal relief—indeed, soon worked out a cure.

It is worthy of remark, that in no stage of this disease was there any tenderness of the spine.

